# Migration of *Escherichia coli* and *Klebsiella pneumoniae* Carbapenemase (KPC)-Producing *Enterobacter cloacae* through Wastewater Pipework and Establishment in Hospital Sink Waste Traps in a Laboratory Model System

**DOI:** 10.3390/microorganisms9091868

**Published:** 2021-09-03

**Authors:** Paz Aranega-Bou, Nicholas Ellaby, Matthew J. Ellington, Ginny Moore

**Affiliations:** 1 Biosafety, Air and Water Microbiology Group, National Infection Service, Public Health England, Manor Farm Rd, Porton Down, Salisbury SP4 0JG, UK; ginny.moore@phe.gov.uk; 2 Antimicrobial Resistance and Health Care Associated Infections, National infection Service, Public Health England, 61 Colindale Avenue, London NW9 5EQ, UK; Nicholas.Ellaby@phe.gov.uk; 3 National Infection Service Laboratories, Public Health England, 61 Colindale Avenue, London NW9 5EQ, UK; Matthew.Ellington@phe.gov.uk

**Keywords:** CPE, drains, hospital plumbing, environmental contamination, infection control

## Abstract

Sink waste traps and drains are a reservoir for multi-drug resistant Gram-negative bacteria in the hospital environment. It has been suggested that these bacteria can migrate through hospital plumbing. Hospital waste traps were installed in a laboratory model system where sinks were connected through a common wastewater pipe. Enterobacterales populations were monitored using selective culture, MALDI-TOF identification and antibiotic resistance profiling before and after a wastewater backflow event. When transfer between sinks was suspected, isolates were compared using whole-genome sequencing. Immediately after the wastewater backflow, two KPC-producing *Enterobacter cloacae* were recovered from a waste trap in which Carbapenemase-producing Enterobacterales (CPE) had not been detected previously. The isolates belonged to ST501 and ST31 and were genetically indistinguishable to those colonising sinks elsewhere in the system. Following inter-sink transfer, KPC-producing *E. cloacae* ST501 successfully integrated into the microbiome of the recipient sink and was detected in the waste trap water at least five months after the backflow event. Seven weeks and three months after the backflow, other inter-sink transfers involving *Escherichia coli* ST5295 and KPC-producing *E. cloacae* ST501 were also observed.

## 1. Introduction

Gram-negative bacteria, in particular, members of Enterobacterales such as *Escherichia coli*, *Klebsiella pneumoniae* and *Enterobacter cloacae* are common inhabitants of the intestinal microbiome and are also among the most common causes of hospital acquired infections [1,2]. Antibiotic resistance among Enterobacterales has become increasingly common in the last 30 years, and has been caused, in large part, by the emergence and plasmid dissemination of enzymes able to degrade β-Lactam antibiotics, such as Extended-Spectrum β-Lactamases and carbapenemases [1]. Carbapenemases are β–lactamase enzymes with the ability to hydrolyse most β-lactam-antibiotics, including carbapenems. Carbapenemases are classified into Ambler class A (e.g., *Klebsiella pneumoniae* carbapenemases (KPC)), class B (e.g., New Delhi metallo-β-lactamase (NDM)) and class D (e.g., β-lactamase OXA-48) [3].

Carbapenem-resistant Enterobacterales have been added to the top priority tier for development of new antibiotics by the World Health Organisation (WHO) [4], as very limited treatment options currently exist and associated mortality can be as high as 53% [5].

Hospital reservoirs for Carbapenemase-producing Enterobacterales (CPE) include colonized patients and wastewater, with contaminated sinks being the most commonly reported environmental reservoir [6]. In fact, shower and sink drains can harbour identical or highly similar strains to patients and are considered a potential source of transmission [7,8,9]. Although bacteria resistant to antibiotics are most often investigated, many sensitive Enterobacterales, including *E. coli*, can also be recovered from sink waste traps [8]. Droplet-mediated transmission during sink flushing activities has been identified as the main mechanism of dispersal from contaminated sinks [10,11]. Importantly, contamination of sink sites might not be localised and might not remain contained. Migration of Gram-negative bacteria through wastewater plumbing to colonize nearby sink and shower drains has been reported [12,13]. Here, we describe the propagation of two strains of KPC-producing *Enterobacter cloacae* and one strain of *Escherichia coli* through pipework in a laboratory model sink system containing hospital waste traps.

## 2. Materials and Methods

### 2.1. Waste Trap Installation in the Laboratory Model System

**Figure 1:**
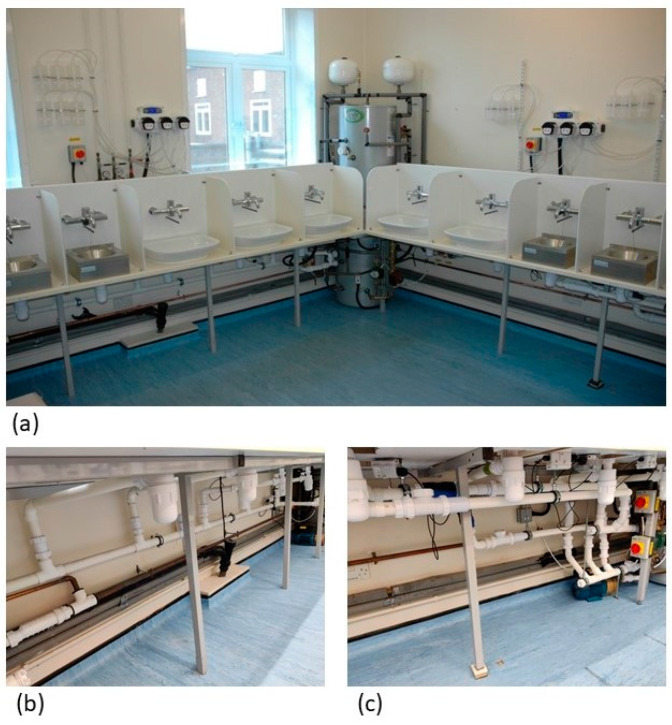
Sinks commonly found in healthcare settings installed as part of a laboratory model system (**a**). Sinks drain into a common wastewater pipe (**b**) or individual wastewater pipes (**c**).

The model system has been described previously [10]. The model incorporates twelve individual sinks with bottle waste traps and associated pipework. Rigid partitions (40 × 57.5 cm) installed between each sink prevent cross-contamination from one sink to another (Figure 1a).

**Figure 2:**
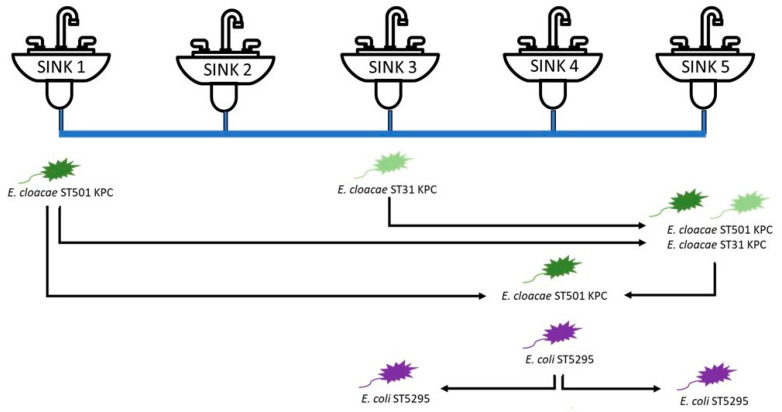
Schematic representation of transfer events involving sinks positioned in line sharing a common wastewater pipe in a laboratory model system.

On January 2018, eight waste traps were collected from a hospital in England, transported to the laboratory and fitted to eight different sinks. Four of these sinks (sinks 1, 3, 4 and 5 in Figure 2) were connected via a common wastewater pipe (Figure 1b) while four drained to individual wastewater pipes (Figure 1c). The waste traps fitted to sinks 1 and 5 were taken from different rooms in the same ward while those fitted to sinks 3 and 4 came from two separate and distinct wards. The remaining sinks (including sink 2 in Figure 2) were fitted with hospital waste traps installed previously (September 2016) and were not monitored as part of this study.

At the time of installation, water and biofilm samples were taken from each waste trap and cultured to enumerate coliform bacteria and CPE using Brilliance™ *E. coli*/coliform agar (Oxoid Ltd., Basingstoke, UK) and chromID™ CARBA medium (Biomérieux, Basingstoke, UK), respectively. All plates were incubated at 37 °C for 18–24 h. Thereafter, approximately 3 mL of nutritious broth was poured into each sink on a daily basis to maintain Enterobacterales populations.

The tap associated with each sink was automatically flushed 4 times a day for 30 s, simulating low usage. Waste trap water populations were regularly monitored using the same methods described above.

### 2.2. Wastewater Backflow Event

During normal operation, water drains from the sinks into a 124 L collecting vessel. Drainage is automatically controlled by means of a pump. Approximately three weeks after the waste traps were installed, normal operation of the pump was impaired. This resulted in wastewater flowing back through common pipework and re-entering the sinks, simulating a major blockage. After remaining stagnant for four hours, the water was drained. After 24 h, the sinks and the taps were allowed to operate again normally. The waste trap water of each sink continued to be collected and cultured on a regular basis. Migration distances were determined by measuring and adding up the pipework length between waste traps involved.

### 2.3. Phenotypical Characterisation of Isolates

Presumptive Enterobacterales were identified using matrix-assisted laser desorption/ionization time-of-flight (MALDI-ToF) mass spectrometry (Bruker Daltonik MALDI biotyper; Bruker, Bremen, Germany) using the direct transfer method. Isolates were compared by antibiotic resistance profiling using the disc diffusion method (ampicillin, gentamicin, amikacin, meropenem, ceftazidime and ciprofloxacin) (Oxoid) following EUCAST guidelines. Isolates were stored in cryobeads (Technical Service Consultants, Heywood, UK) at −80 °C.

### 2.4. Genotypical Characterisation of Isolates

Isolates were recovered from beads and DNA was extracted using QIAamp DNA Mini Kit (Qiagen, Manchester, UK). Whole-genome sequencing (WGS) was carried out on an Illumina HiSeq 2500 (Illumina, San Diego, CA, USA). Reads were submitted to the European Nucleotide Archive (project number PRJEB43840). Genome assemblies were generated by SPAdes [14] and compared using Mash [15]. MLST profile was determined by mapping reads against publicly available species databases (https://pubmlst.org/, accessed on 12 July 2021) using the tool MOST [16]. MLST databases from 2015-06-04 and 2017-04-28 were used to assign sequence types to *Enterobacter cloacae* and *Escherichia coli*, respectively.

## 3. Results

### 3.1. KPC-Producing Enterobacter cloacae Transfer Events

Four hospital waste traps were installed in sinks sharing a common wastewater pipe in a laboratory model system (sinks 1, 3, 4 and 5 in Figure 2). Upon installation, carbapenemase-producing *Enterobacter cloacae* was isolated from sinks 1 and 3. Using disc diffusion assays, it was determined that the strain originating in sink 1 was resistant to ampicillin, ceftazidime and meropenem (Antibiotic resistance profile A). The strain from sink 3 was resistant to those antibiotics and also showed intermediate resistance to ciprofloxacin and gentamicin (Antibiotic resistance profile B). No CPE were recovered from sinks 4 and 5. However, approximately three weeks after the waste traps were installed, a wastewater backflow event occurred resulting in wastewater flowing back through the system and re-entering sinks. Immediately following the backflow event, *E. cloacae* isolates exhibiting antibiotic resistance profiles A and B were detected in the waste trap water of sink 5, having migrated through 327 and 246 cm of pipework, respectively. Subsequent culture results implied that *E. cloacae* with antibiotic resistance profile A had become part of the microbial community colonising the waste trap of sink 5, and isolates were recovered from this sink for at least five months after the initial transfer (Table 1). In contrast, *E. cloacae* with antibiotic resistance profile B was only detected once (i.e., was not detected during subsequent sampling). Approximately 7-weeks after the backflow event, *E. cloacae* isolates exhibiting antibiotic resistance profile A were detected in sink 4, implying another transfer event (Table 1). In this case, bacteria might have migrated from sink 1 (269 cm) and/or from sink 5 (163 cm). Nine isolates recovered from the affected sinks were sequenced and compared. WGS confirmed that two different strains (ST501 originating from sink 1 (antibiotic resistance profile A) and ST31 originating from sink 3 (antibiotic resistance profile B)) had migrated through the system. According to Mash, isolates belonging to ST501 shared over 98.6% similarity within the group, while isolates belonging to ST31 shared over 99.2% (Supplementary Table S1).

### 3.2. Escherichia coli Transfer Events

Upon installation of the waste traps, *E. coli* was detected in sink 4, but none of the other sinks (Table 1). No immediate changes regarding *E. coli* distribution in the system were observed following the backflow event. However, three months later, *E. coli* was detected in the two sinks either side of sink 4 (i.e., sinks 3 and 5) having migrated 190 and 163 cm, respectively. *E. coli* isolates continued to be recovered from sinks 3 and 5 for a further ten weeks and five months, respectively (Table 1). All *E. coli* isolates were sensitive to all antibiotics tested. WGS identified all eight isolates recovered as *E. coli* ST5295 with over 99.4% shared similarity (Supplementary Table S2).

## 4. Discussion

The results presented in this study suggest that CPE contamination within a single waste trap might not remain localised and transfer of Enterobacterales between sinks connected through common pipework is possible and perhaps linked to drainage problems and the backflow of wastewater. Although rarely reported in the literature, plumbing problems such as blockages are frequent in hospitals [17].

Here, we found bacterial transfer occurred between waste traps separated by up to 3.27 metres of pipework. Panels between each sink (Figure 1a) prevented above drain cross-contamination (e.g., via splashback [10]), implicating pipework migration as the most likely pathway. Our system also incorporates sinks that drain to individual wastewater pipes (Figure 1c); bacterial transfer between these sinks has not been observed. Recent work looking into the ecology of *E. coli* and *Klebsiella* spp. populations in hospital waste traps has shown that different wards and even different sinks harbour distinct ecosystems, with few shared lineages [8]. Therefore, it seems unlikely that identical strains were found in different waste traps by chance.

It is interesting to notice that while some transfers were identified immediately after the backflow event (*E. cloacae* ST31 and ST501 from sinks 1 and 3 to sink 5), others were not identified until some months after the event (*E. cloacae* ST501 from sink 1 or 5 to sink 4 and *E. coli* ST5295 from sink 4 to sinks 3 and 5). One possible explanation is that small numbers of bacteria transferred during the backflow were incorporated into the existing biofilm and took time to increase to detectable levels. Another possible explanation is that migrations occurred later and were due to unrelated transfer events.

In a recent study, Hopman et al. [12] described the environmental investigation that followed a fatal hospital-acquired infection cause by a carbapenemase-producing *Pseudomonas aeruginosa*. The same strain was recovered from shower and sink drains in seven different rooms which shared pipework to a common sewage collection point, but not from the sinks and showers in nearby rooms that drained into a different sewage collection point. The authors suggested that the strain was introduced into a drain and then spread via plumbing to proximate rooms. However, as no environmental cultures were available prior to the positive patient cultures, it is unknown when and how the introduction took place and how long it took for it to spread to the other rooms. Other reports have also suggested migration of Gram-negative bacteria through pipework [18,19], but alternative explanations (e.g., introduction via hand-washing) could not be ruled out. A key aspect of the work described in this report is the availability of pre-installation samples (Table 1) which provide information about the bacterial populations before the backflow event.

Some of the transfer events observed in this study led to a stable colonisation lasting for at least 5 months (*E. cloacae* ST501 and *E. coli* ST5295 in sink 5) while in others the colonisation was more transient, lasting from days to a few weeks (*E. cloacae* ST501 in sink 4, *E. cloacae* ST31 in sink 5 and *E. coli* ST5295 in sink 3). It is interesting that a strain that successfully colonized one sink was unable to do so in another, suggesting that the microbial environment can exclude certain strains that could otherwise be successful, or at least prevent their growth to detectable levels. Utilising a similar model sink system, Kotay et al. [13] showed that, in the absence of competing microorganisms, a GFP-expressing *E. coli* strain inoculated on a sink waster trap could be detected in other sinks connected by a common waste pipe, after just a few days. Rapid colonization of newly replaced pipework and sinks with carbapenem-resistant organisms has also been observed in the hospital setting [7]. However, these systems, while not being sterile, lacked the extensive biofilm coverage of old, existing hospital wastewater plumbing. The lack of microbial competition might have facilitated the colonisation of sinks in these scenarios.

## 5. Conclusions

This work supports previous studies that have demonstrated migration of Gram-negative pathogens through plumbing [13] and provides evidence of CPE migration through contaminated wastewater sites. This implies that once a sink is colonised with CPE, the contamination might not remain localised and could spread to other sites via plumbing, particularly when drainage problems and stagnation of wastewater occur.

## Figures and Tables

**Table 1:** Isolates characterised in this study.

	January 2018 (Pre-Installation)	February 2018 (Pre-Backflow)	February 2018 (Post-Backflow)	April 2018	May 2018	August 2018	September 2018	October 2018
SINK 1	*E. cloacae* ST501 (Isolate A)	*E. cloacae* ST501 (Isolate B)						
SINK 2								
SINK 3	*E. cloacae* ST31 (Isolate G)	*E. cloacae* ST31 (Isolate H)			*E. coli* ST5295 (Isolate L)	*E. coli* ST5295 (Isolate N)		
SINK 4	*E. coli* ST5295 (Isolate J)			*E. cloacae* ST501 (Isolate D)	*E. coli* ST5295 (Isolate K)			
SINK 5			*E. cloacae* ST501 (Isolate C) *E. cloacae* ST31 (Isolate I)		*E. cloacae* ST501 (Isolate E) *E. coli* ST5295 (Isolate M)	*E. cloacae* ST501 (Isolate F) *E. coli* ST5295 (Isolate O)	*E. coli* ST5295 (Isolate P)	*E. coli* ST5295 (Isolate Q)

## Data Availability

Sequencing reads are available on the European Nucleotide Archive (number PRJEB43840). MALDI-TOF raw data will be available upon request.

## Supplementary Materials

The following are available online at https://www.mdpi.com/article/10.3390/microorganisms9091868/s1, Table S1: MinHash similarity matrix representing the proportion of kmers shared by the different *Enterobacter cloacae* isolates characterised in this study, Table S2: MinHash similarity matrix representing the proportion of kmers shared by the different *Escherichia coli* isolates characterised in this study.
