# Supplementary material for: Migration of Escherichia coli and Klebsiella pneumoniae Carbapenemase (KPC)-Producing Enterobacter cloacae through Wastewater Pipework and Establishment in Hospital Sink Waste Traps in a Laboratory Model System
Source: Microorganisms. 2021 Sep 3;9(9):1868. doi: 10.3390/microorganisms9091868 (PMC8468231; doi:10.3390/microorganisms9091868)
Supplement: Supplementary file 1 [file microorganisms-09-01868-s001.zip › microorganisms-1314098-supplementary.pdf]

Supplementary Table S1. MinHash similarity matrix representing the proportion of kmers shared by the different *Enterobacter cloacae* isolates characterised in this study.

	**A**	**B**	**C**	**D**	**E**	**F**	**G**	**H**	**I**
**A**	100.0	99.0	99.2	99.1	99.1	98.6	15.9	15.8	15.8
**B**	99.0	100.0	99.4	99.3	99.4	99.0	15.9	15.9	15.9
**C**	99.2	99.4	100.0	99.5	99.6	99.0	15.9	15.9	15.9
**D**	99.1	99.3	99.5	100.0	99.5	99.0	15.9	15.9	15.9
**E**	99.1	99.4	99.6	99.5	100.0	99.2	15.9	15.9	15.9
**F**	98.6	99.0	99.0	99.0	99.2	100.0	15.8	15.8	15.8
**G**	15.9	15.9	15.9	15.9	15.9	15.8	100.0	99.2	99.3
**H**	15.8	15.9	15.9	15.9	15.9	15.8	99.2	100.0	99.7
**I**	15.8	15.9	15.9	15.9	15.9	15.8	99.3	99.7	100.0

Supplementary Table S2. MinHash similarity matrix representing the proportion of kmers shared by the different *Escherichia coli* isolates characterised in this study.

	**J**	**K**	**L**	**M**	**N**	**O**	**P**	**Q**
**J**	100.0	99.9	99.9	99.7	99.9	99.8	99.7	99.5
**K**	99.9	100.0	99.9	99.8	99.9	99.8	99.7	99.6
**L**	99.9	99.9	100.0	99.7	99.8	99.7	99.7	99.5
**M**	99.7	99.8	99.7	100.0	99.8	99.7	99.6	99.4
**N**	99.9	99.9	99.8	99.8	100.0	99.8	99.7	99.6
**O**	99.8	99.8	99.7	99.7	99.8	100.0	99.6	99.5
**P**	99.7	99.7	99.7	99.6	99.7	99.6	100.0	99.4
**Q**	99.5	99.6	99.5	99.4	99.6	99.5	99.4	100.0
